# Supplementary material for: Molecular Diversity of Mycobacterium avium subsp. paratuberculosis in Four Dairy Goat Herds from Thuringia (Germany)
Source: Animals (Basel). 2023 Nov 16;13(22):3542. doi: 10.3390/ani13223542 (PMC10668697; doi:10.3390/ani13223542)
Supplement: Supplementary file 1 [file animals-13-03542-s001.zip › animals-2674041-supplementary.pdf]

animals

MDPI

Supplemented Material

Table S1. *Mycobacterium avium* subsp. *paratuberculosis* isolates (n = 133) from feces of 120 female goats and one buck belonging to herd 1 in Thuringia (Germany) and their respective genotyping results

Sampling	N°	Isolate	Goat N°	Birth year	Genotype [a]
2018	1	18MA0379	01548	2007	T1 [b]
	2	18MA0188	81696	2008	T1
	3	18MA0473	81704	2008	T1
	4	18MA0354	01865	2009	T1
	5	18MA0457	01866	2009	T1
	6	18MA0285	50041	2009	T1
	7	18MA0370	07267	2010	T1
	8	18MA0133	07273	2010	T1
	9	18MA0434	43936	2010	T1
	10	18MA0143	43938	2010	T1
	11	18MA0331	07329	2011	T1
	12	18MA0400	07353	2011	T1
	13	18MA0464	84924	2011	T2 [c]
	14	18MA0440	16984	2012	T1
	15	18MA0323	16988	2012	T1
	16	18MA0345	51580	2013	T1
	17	18MA0338	80938	2014	T1
	18	18MA0123	80947	2014	T1
	19	18MA0114	80951	2014	T1
	20	18MA0118	80956	2014	T1
	21	18MA0363	80958	2014	T1
	22	18MA0317	80962	2014	T1
	23	18MA0383	80966	2014	T1
	24	18MA0364	80986	2014	T1
	25	18MA0318	81002	2014	T1
	26	18MA0409	81014	2014	T1
	27	18MA0462	81027	2014	T1
	28	18MA0337	58000	2015	T1
	29	18MA0321	58002	2015	T1
	30	18MA0280	58011	2015	T1

	31	18MA0155	58020	2015	T1
	32	18MA0439	58024	2015	T1
	33	18MA0420	58029	2015	T1
	34	18MA0456	58033	2015	T1
	35	18MA0303	58034	2015	T1
	36	18MA0394	58038	2015	T1
	37	18MA0190	58039	2015	T1
	38	18MA0113	58043	2015	T1
	39	18MA0402	58052	2015	T1
	40	18MA0112	45803	2016	T1
	41	18MA0092	45828	2016	T1
	42	18MA0152	45832	2016	T1
	43	18MA0171	45893	2016	T1
	44	18MA0101	45915	2016	T1
	45	18MA0385	45927	2016	T1
	46	18MA0427	45932	2016	T1
	47	18MA0357	45945	2016	T1
	48	18MA0088	45969	2016	T1
	49	18MA0347	45985	2016	T1
	50	18MA0090	45993	2016	T1
	51	18MA1444	09142	2017	T1
	52	18MA1406	09149	2017	T1
	53	18MA1403	09173	2017	T1
	54	18MA1467	09194	2017	T1
	55	18MA1482	45859	2017	T1
	56	18MA1448	45902	2017	T1
	57	18MA1457	45921	2017	T1
2020	1	20MA1306	80949	2014	T1
	2	20MA0392	80965	2014	T1
	3	20MA0472[e]	80967	2014	T3 [d]
	4	20MA0979[f]	80967	2014	T3
	5	20MA1199	80975	2014	T1
	6	20MA0288	80976	2014	T1
	7	20MA0991	80984	2014	T1
	8	20MA1011	81014	2014	T1
	9	20MA1327	81024	2014	T2
	10	20MA0362	58031	2015	T1

	11	20MA0424	58041	2015	T1
	12	20MA0282	58055	2015	T1
	13	20MA0999	58056	2015	T1
	14	20MA1614	59546*	2015	T1
	15	20MA1171	45800	2016	T1
	16	20MA0328	45809	2016	T1
	17	20MA1012	45898	2016	T1
	18	20MA0975	45917	2016	T1
	19	20MA0343	<u>45931</u>	2016	T1
	20	20MA1257	<u>09166</u>	2017	T1
	21	20MA0377	45842	2017	T1
	22	20MA0206	45855	2017	T1
	23	20MA1293	<u>45990</u>	2017	T1
	24	20MA0345	72012	2018	T1
	25	20MA0273	72026	2018	T1
	26	20MA0348	72027	2018	T1
	27	20MA1282	72038	2018	T1
	28	20MA0326	72073	2018	T1
	29	20MA0231	72051	2018	T1
	30	20MA0975	72073	2018	T1
	31	20MA0292	72081	2018	T1
	32	20MA0417	72086	2018	T1
	33	20MA1467	72101	2018	T1
	34	20MA0208	72126	2018	T1
	35	20MA0224	72133	2018	T1
	36	20MA1428	72437	2019	T1
	37	20MA1262	72474	2019	T1
	38	20MA1484	17307	2020	T1
	39	20MA1606	17344	2020	T1
2021	1	21MA1548	<u>80949</u>	2014	T1
	2	21MA0066	81013	2014	T1
	3	21MA0054	<u>81024</u>	2014	**T2**
	4	21MA0146	58004	2015	T1
	5	21MA1567	58021	2015	T1
	6	21MA1556	58023	2015	T1
	7	21MA0115	58032	2015	T1
	8	21MA0744	<u>58039</u>	2015	T1

	9	21MA1513	45828	2016	T1
	10	21MA1557	45880	2016	T1
	11	21MA0145	45883	2016	T1
	12	21MA0124	45899	2016	T1
	13	21MA1582	45971	2016	T1
	14	21MA1521	09149	2017	T1
	15	21MA0028	09153	2017	T1
	16	21MA0584	09166	2017	T1
	17	21MA0123	09170	2017	T1
	18	21MA0755	13766	2017	T1
	19	21MA0098	13770	2017	T1
	20	21MA0849	45859	2017	T1
	21	'21MA0060	45903	2017	T1
	22	21MA0540	45931	2017	T1
	23	21MA0681	45939	2017	T1
	24	21MA0805	45964	2017	T1
	25	21MA1505	45982	2017	T1
	26	21MA2262	72059	2018	T1
	27	21MA0857	72192	2019	T1
	28	21MA2086	17389	2020	T1
	29	21MA2214	17406	2020	T3
2022	1	22MA0971	45990	2017	T1
	2	22MA1102	72025	2018	T1
	3	22MA1969	72028	2018	T1
	4	22MA2038	72099	2018	T1
	5	22MA1148[g]	72120	2018	T1
	6	22MA2046[h]	72120	2018	T1
	7	22MA2161	17338	2020	T1
	8	22MA2101	17423	2020	T1

[a] Genotype of isolates comprises combined of results of three typing methods: (1) mycobacterial interspersed repetitive unit-variable number of tandem repeat (MIRU-VNTR)-typing according to [40] with resulting profiles designated according to INRA Nouzilly MIRU-VNTR (INMV) nomenclature deposited in the INMV database (http://mac-inmv.tours.inra.fr), (2) short sequence repeat (SSR) analysis according to [63] with profiles representing the repeat numbers at specific target sites of G1 (SSR1), GGT (SSR8), and TGC (SSR9), and (3) SNP based assay according to [38] resulting in assignment of strain to phylogenetic groups; [b] Genotype T1 includes profile: INMV1 / 7-4-4 / Clade 9; [c] Genotype T2 includes profile: INMV33 / 7-4-4 / Subgroup B; [d] Genotype T3 includes profile: INMV2

/ 7-4-4 / Clade 1; * Buck; Underlined – goats (n = 12) of which two isolates were genotyped. [e] isolated in April 2020, [f] isolated in July 2020, [g] isolated in May 2022, [h] isolated in November 2022

Table S2. *Mycobacterium avium* subsp. *paratuberculosis* isolates of goats and their environment from different Thuringian herds (herd 2 to 4) and their respective genotypes

Herd	Sampling	Isolate	Origin	Genotype [a]
**2**	2019	19MA0520	Feces	T4 [b]
		19MA0523	Feces	T4
		19MA0524	Feces	T4
		19MA0541	Feces	T4
		19MA0552	Feces	T4
	2022	22MA1240	Feces	T4
		22MA0076	Feces	T4
**3**	2017	<u>17MA0621</u>	Feces	T5 [c]
		<u>17MA0656</u>	Feces	T5
	2022	22MA0039	Feces, bulk sample	T5
**4**	2017	17MA0283	Feces	T6 [d]
		17MA0699	Feces	T6
		17MA0704	Feces	T6
		17MA1022	Bedding	T6
		17MA1023	Bedding	T6
		17MA1024	Bedding	T6
		17MA1025	Bedding	T6
		17MA1026	Bedding	T6
		17MA1034	Bedding	T6
	2020	<u>20MA0040</u>	LNN mesenteriales	T6
		<u>20MA0041</u>	Jejunum	T6
	2021	21MA1973	Feces, bulk sample	T6

[a] Genotype of isolates comprises combined of results of three typing methods: (1) mycobacterial interspersed repetitive unit-variable number of tandem repeat (MIRU-VNTR)-typing according to [40] with resulting profiles designated according to INRA Nouzilly MIRU-VNTR (INMV) nomenclature deposited in the INMV database (http://mac-inmv.tours.inra.fr), (2) short sequence repeat (SSR) analysis according to [63] with profiles representing the repeat numbers at specific target sites of G1 (locus1), GGT (locus 8), and TGC (locus 9), and (3) SNP based assay according to [38] resulting in assignment to phylogenetic groups. [b] Genotype T4 includes profile: INMV2 / 7-4-5 / Clade 4. [c] Genotype T5 includes profile: INMV3 / 7-4-4 / Clade 4. [d] Genotype T6 includes profile:

INMV16* / 7-4-4 / Clade 3. INMV16* is characterized by a specific sequence at MIRU-VNTR locus VNTR 7, explained in the paper. Underlined – isolates originated from the same goat; all other isolates from feces or tissues originated from a different goat. LNN – Lymphonodus.

Table S3. *Mycobacterium avium* subsp. *paratuberculosis* isolates (n = 34) from different tissues of 13 goats belonging to herd 1 in Thuringia (Germany) and their respective genotyping results.

Goat N°	Birth year	Isolate	Origin	Genotype [a]
80940	2014	18MA0229	Jejunum	T1 [b]
80949	2014	22MA0306	Spleen	T1
80949	2014	22MA0311	LNN ileocolici	T1
80949	2014	22MA0319	LNN inguinales superficiales	T1
80949	2014	22MA0320	Jejunum	T1
80974	2014	18MA0266	LNN ileocolici	T1
80993	2014	18MA0241	Ileum	T1
80993	2014	18MA0242	LNN mesenteriales	T1
81014	2014	20MA1134	Jejunum	T1
81014	2014	20MA1142	LNN colici	T1
57999	2015	18MA0199	Jejunum	T1
57999	2015	18MA0204	LNN ileocolici	T1
58007	2015	18MA0210	Ileum	T1
58007	2015	18MA0213	LNN ileocolici	T1
58007	2015	18MA0214	Mammary gland	T1
58049	2015	18MA0250	Jejunum	T1
58049	2015	18MA0252	Ileum	T1
58049	2015	18MA0255	LNN ileocolici	T1
58050	2015	18MA0219	Jejunum	T1
58050	2015	18MA0221	Ileum	T1
58050	2015	18MA0224	LNN ileocolici	T1
58056	2015	20MA1146	Jejunum	T1
58056	2015	20MA1147	Jejunum	T1
45859	2017	22MA1383	Jejunum	T1

45859	2017	22MA1385	Ileum	T1
45859	2017	22MA1387	LNN mesenteriales	T1
45859	2017	22MA1388	LNN ileocolici	T1
45925	2017	22MA0191	Jejunum	T1
45925	2017	22MA0199	LNN mesenteriales	T1
45925	2017	22MA0204	LNN hepatici	T1
72073	2018	22MA1395	Jejunum	T1
72073	2018	22MA1398	LNN mesenteriales	T1
72073	2018	22MA1400	LNN ileocolici	T1
72073	2018	22MA1404	LNN hepatici	T1

LNN - Lymphonodi; [a] Genotype of isolates comprises combination of results of three typing methods: (1) mycobacterial interspersed repetitive unit-variable number of tandem repeat (MIRU-VNTR)-typing according to [40] with resulting profiles designated according to INRA Nouzilly MIRU-VNTR (INMV) nomenclature deposited in the INMV database (http://mac-inmv.tours.inra.fr), (2) short sequence repeat (SSR) analysis according to [63] with profiles representing the repeat numbers at specific target sites of G1 (locus1), GGT (locus 8), and TGC (locus 9), and (3) SNP-based assay according to [38] resulting in assignment of strains to phylogenetic groups; [b] Genotype T1 includes profile: INMV1 / 7-4-4 / Clade 9.

Table S4. *Mycobacterium avium* subsp. *paratuberculosis* isolates (n = 29) from environmental samples of herd 1 in Thuringia (Germany) and their respective genotyping results

**Isolate**	**Sampling N°***	**Localization**	**Sample material**	**Genotype [a]**
20MA0563	1	Kidding area	Dust	T1 [b]
20MA0580	1	Kidding area	Bedding	T1
20MA0590	1	Milking parlor	Bedding	T1
20MA0591	1	Waiting area milking parlor	Bedding	T1
20MA1027	1	Stable entrance front	Bedding	T3 [c]
20MA1028	1	Stable entrance back	Bedding	T1
20MA1706	2	Kidding area	Bedding	T1
20MA1707	2	Kidding area	Bedding	T1
20MA1708	2	Kidding area	Bedding	T1
21MA0859	3	Milking parlor	Bedding	T1
21MA0862	3	Kidding area	Bedding	T1
21MA0863	3	Kidding area	Bedding	T1
21MA0864	3	Kidding area	Bedding	T1
21MA0865	3	Kidding area	Bedding	T1

21MA2612	5	Waiting area milking parlor	Bedding	T1
21MA2613	5	Milking parlor	Bedding	T1
21MA2614	5	Exit area milking parlor	Bedding	T1
21MA2615	5	Stable quadrant 1	Bedding	T1
21MA2616	5	Stable quadrant 2	Bedding	T1
22MA0157	6	Waiting area milking parlor	Bedding	T1
22MA0158	6	Milking parlor	Bedding	T1
22MA0160	6	Stable quadrant 1	Bedding	T1
22MA0161	6	Stable quadrant 2	Bedding	T1
22MA0162	6	Stable quadrant 3	Bedding	T1
22MA0163	6	Stable quadrant 4	Bedding	T1
22MA0700	7	Milking parlor	Bedding	T1
22MA0704	7	Stable quadrant 3	Bedding	T1
22MA1350	8	Milking parlor	Bedding	T1
22MA1353	8	Stable quadrant 2	Bedding	T1

[a] Genotype of isolates comprises combined of results of three typing methods: (1) mycobacterial interspersed repetitive unit-variable number of tandem repeat (MIRU-VNTR)-typing according to [40] with resulting profiles designated according to INRA Nouzilly MIRU-VNTR (INMV) nomenclature deposited in the INMV database (http://mac-inmv.tours.inra.fr), (2) short sequence repeat (SSR) analysis according to [63] with profiles representing the repeat numbers at specific target sites of G1 (locus1), GGT (locus 8), and TGC (locus 9), and (3) SNP based assay according to [38] resulting in assignment of strains to phylogenetic groups. [b] Genotype T1 includes profile: INMV1 / 7-4-4 / Clade 9. [c] Genotype T3 includes profile: INMV2 / 7-4-4 / Clade 1. Details about environmental sampling were described elsewhere [12].
